# Suicide mortality in eastern and western Germany, 1952–2022: Construction and validation of time series using joinpoint models with jump detection

**DOI:** 10.1371/journal.pone.0349502

**Published:** 2026-06-17

**Authors:** Enno Nowossadeck, Claudia Hövener, Niels Michalski

**Affiliations:** 1 Department of Epidemiology and Health Monitoring, Robert Koch Institute, Berlin, Germany; 2 University of Applied Sciences -Alice-Salomon-Platz 5, Berlin, Germany; Facultad Latinoamericana de Ciencias Sociales Mexico, MEXICO

## Abstract

Suicide mortality is a significant public health problem. Retrospective analyses can be helpful in better understanding temporal trends of suicide mortality and thus derive clues about causal and associated factors for the course. For this paper, we compiled suicide mortality rates for eastern and western Germany from 1952 to 2022 from various data sources, stratified by sex. The period encompasses events that could substantially alter the statistically recorded rates, such as the changes in versions of the International Classification of Diseases (ICD) in 1978 and 1997, but also the integration of the statistical system of the German Democratic Republic (GDR) into that of the Federal Republic of Germany (FRG) in 1990/91. These could cause methodologically induced breaks in the compiled time series. Using Joinpoint-Jump models, we tested whether at these defined points a jump, i.e., an abrupt change in the level of the time series, can be statistically detected or rejected. The empirical results show a high dynamism of suicide mortality in both parts of Germany, with very frequent increases and decreases of suicide mortality rates. However, the analyses do not point to systematic and substantial methodologically induced structural breaks resulting from ICD version changes or from transferring of the GDR statistical system to that of the FRG. The analysis results support the conclusion that the compiled long time series for eastern and western German women and men are largely consistent. It is unlikely that analyses of trends in suicide rates in Germany during that period are distorted by methodological artefacts examined. Some caution is warranted for women during periods of ICD code changes, although any methodological effects are likely small and within the range of typical annual fluctuations.

## Introduction

Suicides are a major public health problem. Analyses of the World Mental Health (EU-WMH) survey in Germany revealed a lifetime prevalence of suicide ideation of 9.7% among adults, 2.2% for suicide plans and 1.7% for suicide attempts [1,2]. A recent study found an average lifetime prevalence of suicidal thoughts of 7.8% for the period 2003–2020 [3], with no significant differences measured between women and men. Since 1990, almost 360,000 suicide deaths have been registered in Germany – approximately 80% in western Germany, and around 20% in eastern Germany [4]. Small-scale differences and temporal dynamics exist within the two regions [5]. At the time of German reunification in 1990, suicide mortality in eastern Germany was considerably higher than in western Germany and in many other European countries [6–8]. In the following years, suicide mortality declined in both parts of Germany, and this continued until around 2007 [9]. In view of this temporal dynamic and unclear causal mechanisms, a retrospective analysis of suicide mortality in Germany is of great interest. However, the availability of consistent time-series data is a mandatory prerequisite for such an analysis. The existence of methodologically induced structural breaks in the periods to be analysed would represent a problem to be taken into account in the analysis. One example of this is the version changes to the International Classification of Diseases (ICD). Each revision can cause breaks in the comparability of the data [10], and this can have a negative impact on the consistency of the time series of the analysed causes of death.

Another factor that can impact on the consistency of the time series is the transfer of the official statistics system of the German Democratic Republic (GDR) to that of the Federal Republic of Germany (FRG) in the course of reunification in 1990. Starting in October 1990, the statistical system in eastern Germany was structurally and conceptually reorganized and standardized for the whole of Germany. Thus, data sets on the cause-of-death statistics for eastern and western Germany, collected according to standardized regulations and guidelines, have been available for analysis since 1991 [11]. As pertinent analyses have shown, there were also different coding practices and customs in the GDR and the FRG despite their using the same ICD versions [12].

This shows that methodological consistency of the statistical time-series data required for trend analyses – including the reunification period – should not be assumed *a priori*. The problems of integrating GDR statistics into those of the FRG have been described for some statistical indicators, for example for national accounts [13]. Although previous studies have analysed suicide trends in Germany for selected time periods [6,14], a comprehensive assessment covering the full observation period and explicitly addressing potential artefacts such as ICD revisions and reunification-related changes appears to be lacking.

To assess the usability of the data for analysing temporal trends, this paper pursues the following objectives:

a to merge time series for eastern and western Germany for the period from 1952 to 2022 from various data sources, and
b to check for any methodologically induced structural breaks and to ensure that no such breaks in time series exist in future analyses.

## Materials and methods

### Data

For the analysis, data from the national cause-of-death statistics of the GDR and the FRG were used. The compilation and coding of suicide deaths in the cause-of-death statistics is standardized internationally using the ICD. This coding system, developed by the WHO, has been the basis of the statistical systems of the GDR and the FRG in the latest versions of cause-of-death documentation since the 1950s.

The ‘European Shortlist for Causes of Death’ published by Eurostat is available for historical comparisons. It combines the deaths from groups of diseases and external causes in the ICD versions 8, 9 and 10 [15]. In the case of suicide deaths, the category is called ‘Suicide and intentional self-harm’.

When the official statistics system of the GDR was transferred to that of the FRG in 1990/1991, data from the cause-of-death statistics for previous years were also transferred. These can be retrieved retrospectively up to 1980 from the online health-reporting database (https://www.gbe-bund.de/gbe). For the analyses, further data for the period from 1952 to 1979 were compiled from various sources. For the FRG, data is available from the 1952–1979 Statistical Yearbooks [16]. Data on suicide deaths were collected annually in the GDR; however, they were systematically withheld from public release starting in 1972–1989 [6,17,18] (cf. Table 1).

**Table 1:** Compilation of sources for eastern and western Germany in different time periods.

Period	Eastern Germany	Western Germany
1952-1979	GESIS Archive Research Data [19]	Statistical yearbooks [16]
1980-2022	Online database for health reporting [4]	Online database for health reporting [4]

Source: own compilation.

GDR data for the period 1952–1979 were included in the present analyses. It therefore seems appropriate to examine whether the change of data source in 1979/1980 introduced a methodologically induced structural break, since the possibility of incomplete datasets cannot be excluded.

A methodologically induced structural break is defined as a discontinuity in a time series that results from changes in data compilation, coding, or aggregation procedures. These changes alter the measurement basis of the series and create a shift in level and/or trend that is not attributable to the underlying phenomenon. Such discontinuities or ‘jumps’ in the data can potentially lead to misleading interpretations if they are not taken into account [20].

There may be methodologically induced structural breaks in the suicide mortality data for several year pairs. Table 2 shows these year pairs, keeping eastern and western Germany separate; for the period 1952–1990, the terms GDR and FRG are used, while for 1991–2022 the terms new and old federal states are used.

**Table 2:** Timing of possible methodologically induced structural breaks.

East		West	
Year	Event	Year	Event
1977/78	Version switch from ICD8 to ICD9	1977/78	Version switch from ICD8 to ICD9
1979/80	Change in the data source		
1990/91	Reorganization of official statistics		
1997/98	Version switch from ICD9 to ICD10	1997/98	Version switch from ICD9 to ICD10

Source: own compilation.

The analyses included the crude rates of suicide mortality (deaths per 100,000 of the population) for eastern and western Germany, stratified by sex. Although the change of the age composition of the population changes over time, it does so only gradually. Therefore, we do not expect this to affect the validity of our analyses, which are mainly concerned with abrupt changes in trends.

### Hypotheses

This article examines whether there are methodologically induced structural breaks. The overarching hypothesis H is therefore as follows: there are no methodologically induced structural breaks in the sex-specific time series for eastern and western Germany at the points in time mentioned in Table 2. The following hypotheses are tested for the relevant groups in separate statistical analyses:

**Hypothesis H1a.** The first hypothesis is that there is no methodologically induced structural break in 1977/78 due to the respective ICD version change.

**Hypothesis H1b.** Analogous to H1a, it was assumed that there is no methodologically induced structural break in 1997/98.

**Hypothesis H2.** The second hypothesis assumes that there is no methodologically induced structural break in the GDR time-series data for 1979/80 as a result of the change in data access.

**Hypothesis H3.** The third hypothesis is that there is no methodologically induced structural break in the time-series data for 1990/91 as a result of the reorganization of official statistics in eastern Germany.

### Methods

The suicide rates were retrieved separately for women and men from the above-mentioned databases and collated for FRG/GDR and west/east respectively. This resulted in four time series: women in western and in eastern Germany and men, also in western and eastern Germany.

Joinpoint-jump model calculations were carried out for the compiled time series. The joinpoint analysis is based on the idea of locating, estimating and visualizing trends in time series through a sequence of connected linear segments. For each number of joinpoints (or linear segments), the procedure tests all possible joinpoint positions within the data and selects the model with the best fit using the Bayesian Information Criterion [21]. The best-fitting solution for each number of joinpoints is reported to illustrate the range of plausible models and avoid overinterpretation of a single specification.

In addition, joinpoint-jump models estimate whether there is a sudden jump within one of the linear trend segments identified by the joinpoint regression, resulting, for example, from a change in health-statistics coding [20]. Models were calculated for the year pairs specified in Table 2. So-called ‘comparability ratios’ (CRs) were calculated to statistically assess whether the jumps indicate a break in the respective time series. CRs represent the ratio of rates estimated by the linear trend before and after the jump [20]. A CR of 1 indicates that the rates are unaffected by the method change (such as an ICD version change, etc.), meaning no methodologically induced structural break is present. A CR greater than 1 indicates that the rates after the method change are higher than before, while a CR less than 1 indicates that the rates after the method change are lower after the change. P-values were calculated for each CR to test whether the jump represents a statistically significant change (i.e., whether CR differs from 1). P-values for the comparability ratios were calculated using Wald tests based on the unconstrained standard error of the estimated jump parameter (testing H_0_: CR = 1) [20].

Thus, the joinpoint-jump models can identify potential methodologically induced structural breaks. However, accurate inference requires that the assumed jump does not occur near or at the end of a segmented trend, as this can lead to confusion between the joinpoint and the jump. It should be noted that a jump can only be identified within a time interval between two joinpoints. However, as the number of joinpoints changes across models, the time periods defined by the joinpoints can also change. The models are only comparable with each other with regard to the jumps if the time periods in which the jump is assumed do not differ significantly between models.

To ensure the validity of the substantive analysis, it is necessary to test the hypothesis that the data are free of methodologically induced structural breaks. If there are no such methodologically induced structural breaks, it can be assumed that the compiled time series are consistent. To validate the result, we tested for type II errors (beta errors). This refers to failing to reject the presence of jumps based on the test statistics, even when a jump is actually present. To this end, we applied a statistical power of 80% in line with [22]: the hypothesis should not be rejected if the p-value is greater than 0.20. Results with p-values larger than 0.05 and less than 0.20 thus remain inconclusive.

The maximum number of seven joinpoints follows the recommendation to select seven joinpoints as the default setting for a time-series length of at least 37 points in time [23]. 71 points in time are available in each of the time series analysed. In the results tables, the results for two to seven joinpoints are shown for all models to gain a deeper understanding; in the figures and in the discussion, the results of the models with seven joinpoints were used.

This procedure is used to check whether there are significant values for the CRs, i.e., significant jumps, for the specified year pairs. A methodologically induced structural break is assumed if the following criteria apply:

a) The prerequisite for testing is that the time point under examination for the time series falls approximately within the same trend segment.
b) All jumps move in the same direction for one point in time that is to be tested, i.e., all relevant comparability ratios of a point in time are either greater than 1 (increase) or less than 1 (decrease).
c) The comparability ratios are significant for the respective point in time in all populations studied (women/men x eastern/western Germany).
d) The hypothesis that there is no significant jump cannot be rejected (test beta error).

## Results

**Fig 1:**
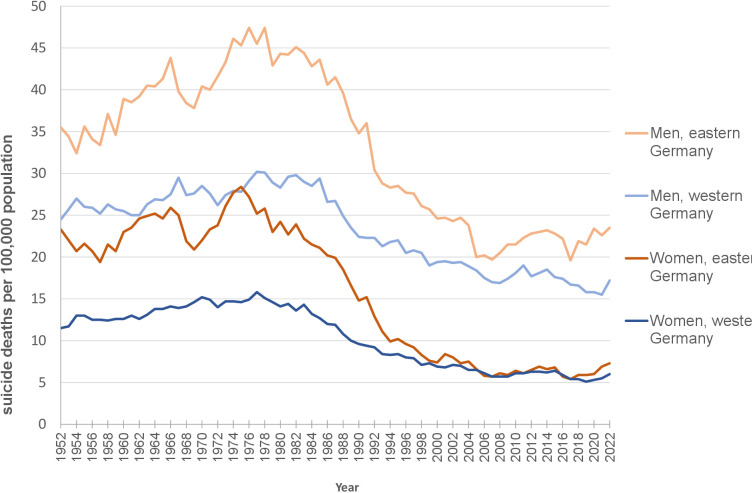
Suicide mortality in eastern and western Germany 1952-2022: presentation of the compiled time series, suicide deaths per 100,000 of the population, women and men.

Combining the data from the different data sources makes time series of crude suicide-mortality rates available for the period from 1952 to the present. These are shown graphically in Fig 1.

Suicide mortality in eastern Germany is higher than in western Germany, for both women and men. However, since the turn of the millennium, there have been no – or only slight – differences between women in the two parts of the country. Over the entire period, the suicide mortality rate is higher among men than among women.

Ignoring annual fluctuations, the temporal development can be divided into three phases with distinct quantitative characteristics:

- Phase I: up until the first half of the 1980s, suicide mortality rates rose among both women and men.
- Phase II: this was followed by a decline in suicide mortality rates in both parts of Germany and among both women and men, which continued into the first half of the 2000s.
- Phase III: a plateau phase began around the mid-2000s.

The decline during Phase II was greater in eastern Germany than in the western part, for both women and men.

Tables 3–6 contain the results of the joinpoint-jump models with two to seven joinpoints for each of the year pairs shown in Table 2. As a rule, the models with two joinpoints reflect the three phases (with the exception of men in western Germany), thus indicating the phase in which the jump is empirically located. The joinpoint-jump models are discussed separately for the year pairs below.

**Table 3:** Linear segment of the JP model in which the jump of the 1977/78 year pair falls, and the corresponding CR.

Number of	East						West					
Joinpoints	Women			Men			Women			Men		
	Trend segment	CR	p-value of the CR	Trend segment	CR	p-value of the CR	Trend segment	CR	p-value of the CR	Trend segment	CR	p-value of the CR
2	1952-1982	0.914	0.099	1952-1982	0.950	0.130	1952-1981	0.918	0.009	1952-1985	0.994	0.786
3	1952-1983	0.923	0.093	1952-1983	0.950	0.097	1952-1981	0.933	0.022	1952-1985	0.993	0.730
4	1976-1987	1.023	0.805	1952-1982	0.951	0.114	1952-1978	0.982	0.450	1952-1985	0.994	0.755
5	1976-1987	1.023	0.796	1952-1983	0.970	0.073	1952-1981	0.932	0.003	1952-1985	0.994	0.738
6	1976-1987	1.023	0.780	1975-1987	1.013	0.774	1977-1983	0.972	0.386	1952-1985	0.993	0.692
7	1975-1988	1.014	0.828	1976-1987	1.031	0.546	1977-1983	0.978	0.484	1967-1985	1.023	0.406

CP - Comparability Ratio, JP – Joinpoint.

**Table 4:** Linear segment of the JP model in which the jump of the 1979/80 year pair falls, and the corresponding CR.

Number of	East					
Joinpoints	Women			Men		
	Trend segment	CR	p-value of the CR	Trend segment	CR	p-value of the CR
2	1978-2006	1.128	0.220	1952-1982	0.952	0.225
3	1952-1985	0.896	0.016	1952-1985	0.917	0.003
4	1976-1988	1.073	0.388	1952-1982	0.953	0.298
5	1976-1988	1.073	0.371	1952-1984	0.931	0.013
6	1976-1988	1.074	0.335	1976-1987	1.023	0.656
7	1975-1988	1.067	0.341	1977-1987	1.024	0.623

CP - Comparability Ratio, JP – Joinpoint.

**Table 5:** Linear segment of the JP model in which the jump of the 1990/91 year pair falls, and the corresponding CR.

Number of	East					
joinpoints		Women			Men	
	Trend segment	CR	p-value of the CR	Trend segment	CR	p-value of the CR
2	1980-2006	0.901	0.078	1981-2006	0.924	0.036
3	1980-2006	0.892	0.048	1983-2006	0.925	0.038
4	1988-1994	1.160	0.300	1981-2007	0.928	0.028
5	1988-1994	1.163	0.270	1983-2006	0.925	0.026
6	1988-1994	1.158	0.252	1988-1993	1.110	0.298
7	1988-1994	1.160	0.213	1988-1993	1.121	0.229

CP - Comparability Ratio, JP – Joinpoint.

**Table 6:** Linear segment of the JP model in which the jump of the 1997/98 year pair falls, and the corresponding CR.

Number of	East						West					
joinpoints	Women			Men			Women			Men		
	Trend segment	CR	p-value of the CR	Trend segment	CR	p-value of the CR	Trend segment	CR	p-value of the CR	Trend segment	CR	p-value of the CR
2	1980-2006	1.008	0.895	1982-2006	1.067	0.116	1994-2022	0.881	0.000	1990-2022	0.948	0.015
3	1982-1999	0.962	0.676	1985-2005	1.851	0.075	1994-2020	0.898	0.001	1990-2020	0.956	0.045
4	1994-2007	0.951	0.490	1982-2007	1.066	0.100	1978-2007	1.034	0.245	1989-2008	1.002	0.949
5	1994-2007	0.939	0.358	1985-2005	1.085	0.056	1993-2008	0.953	0.140	1989-2008	1.003	0.920
6	1994-2007	0.958	0.508	1993-2006	1.023	0.638	1993-2008	0.958	0.145	1990-2004	0.969	0.339
7	1994-2007	0.951	0.407	1994-2003	0.944	0.288	1993-2003	0.903	0.008	1989-2008	1.003	0.895

CP - Comparability Ratio, JP – Joinpoint. Source: own calculations; model selection method: BIC3; maximum number of joinpoints: 7.

Data sources: [4,16,19].

### 1977/78 year pair

**Fig 2:**
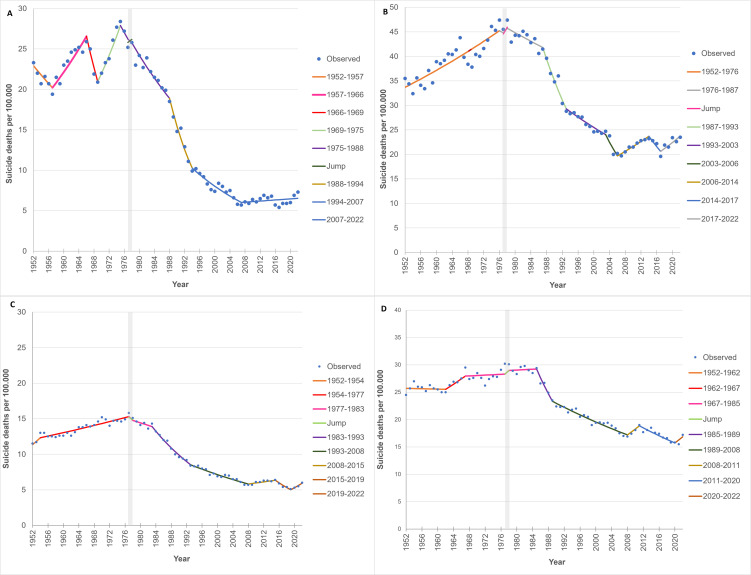
Results of the joinpoint-jump modellings, 1977/78. A) women, eastern Germany, B) men, eastern Germany, C) women, western Germany, D) men, western Germany.

The 1977/78 year pair lies in the final temporal section of Phase I (see Fig 2A-2D). Depending on the number of permitted joinpoints, the joinpoint-jump models identify two different trend segments into which the year pair falls (see Table 3).

In models with a small number of joinpoints, the year pair falls into the trend segment from 1952 to 1981/85 thus covering the entirety of Phase I. Models with more joinpoints identify a shorter segment extending from the mid-1970s to the mid-1980s. In this latter segment, the p-values of the CR for the jump exceeds 0.20 across all subgroups. No statistically significant jump is detected.

In models in which the trend segment covers the entire Phase I, the CR is smaller than 1. Significant jumps only occur in two cases: the models with 2 and 5 joinpoints for women in the west. In several additional cases (women east: 2, 3 joinpoints, men east: 2–5 joinpoints, women west: 3 joinpoints), the null hypothesis of the absence of jumps cannot be rejected (p > 0.05 and p < 0.2). For men in the west, the rejection of a jump is unambiguous (p > 0.20).

Although the hypothesis of a jump cannot be rejected in some specifications, there is no evidence of a consistent jump over all subgroups. With the exception of men in the west, suicide rates peak around 1977 (see Fig 2A-2D), which complicated the identification of a jump at that time, as the estimated drop may reflect the start of the underlying downward trend in suicide rates. For men in the west, where the decline in suicide rates occurred later (Fig 2D) and the test for the jump is therefore more informative, the CR exceeds 1 and remains statistically insignificant.

### 1979/80 year pair

**Fig 3:**
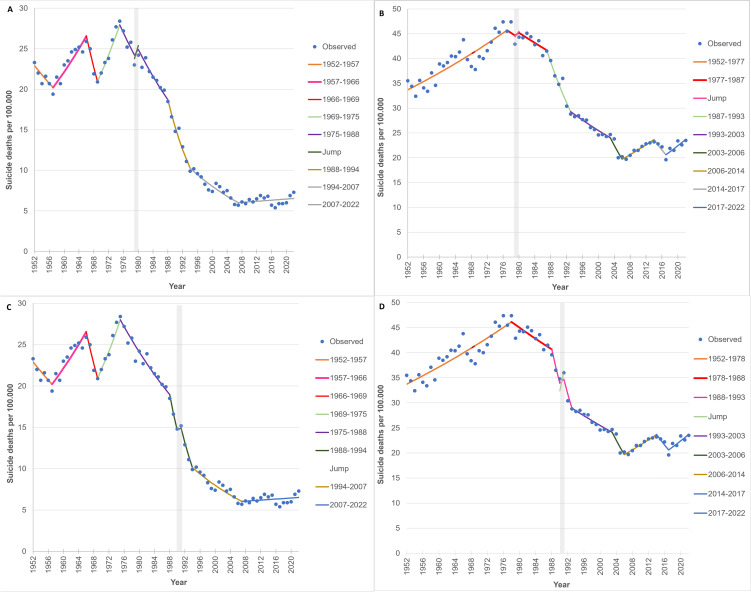
Results of the joinpoint-jump modellings, 1979/80 and 1990/91. A) women, eastern Germany, B) men, eastern Germany. C) women, eastern Germany, D) men, eastern Germany.

The 1979/80 year pair also falls within the final section of Phase I and yields similar results to those for the 1977/78 year pair (Fig 3A-3B). Depending on the number of permitted joinpoints, the models identify either a long trend segment between 1952–1982/85 or a shorter segment from 1975/77–1987/88. In the shorter segment, none of the estimated jumps are statistically significant, thus no jump is detected (women: 2, 4–6 joinpoints, men: 6 and 7 joinpoints). Models with fewer joinpoints tend to identify a segment covering the entire Phase I (1952–1982/85), specifically for men (2–5 joinpoints) and women (3 joinpoints).

Although we cannot reject the hypothesis of a jump in some specifications, there is no consistent evidence of a jump across the subgroups. Overall, there is no clear statistical evidence of systematic or robust jumps related to the 1979/80 year pair.

### 1990/91 year pair

This year pair falls within Phase II, which is characterized by declining suicide mortality rates in all populations (Fig 3C-3D). Two trend segments are identified: a longer segment extending from the early 1980s to the mid-2000s (women 2–3: joinpoints; men: 2–5 joinpoints), and a shorter segment covering the period from 1988 to 1993/94 (women: 4–7 joinpoints, men: 6–7 joinpoints) (see Table 5).

For the segments beginning in 1980/81, the estimated jumps are associated with a decline in suicide mortality (CR < 1) and are statistically significant (women 2–3 joinpoints, men 2–5 joinpoints). In contrast, no significant jumps are observed for the shorter segments beginning in 1987/88. Moreover, the corresponding p-values are far from conventional significance thresholds (p > 0.20), suggesting that the absence of significance is not due to insufficient statistical power.

The evidence for a methodologically induced structural break is therefore mixed. However, an inspection of the graphs indicates that models with a higher number of joinpoints are preferable, as these segments begin at or shortly after the sharp decline marking the onset of Phase II. In contrast, segments that start earlier, when the decline is less pronounced, or that extent into periods where the trend flattens may distort the estimation of a linear decline across Phase II (cf. Fig 3C-3D). Hence, a jump for this date is unlikely.

### 1997/98 year pair

**Fig 4:**
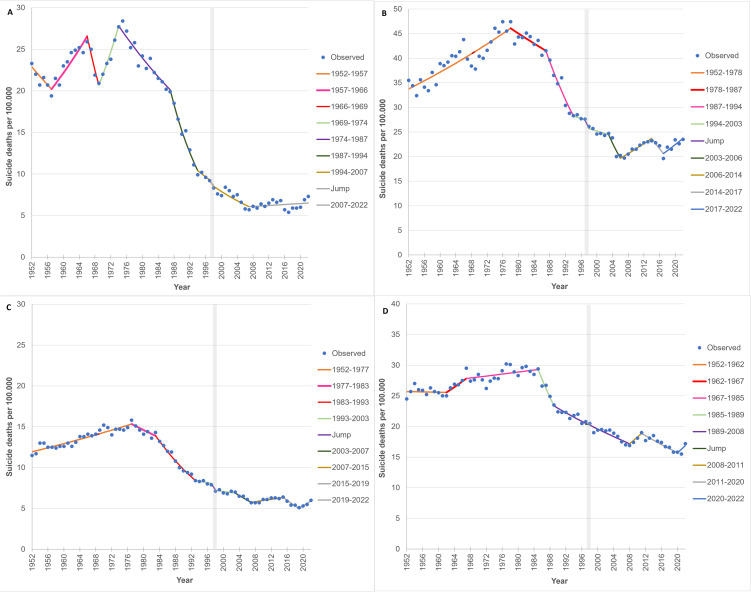
Results of the joinpoint-jump modellings, 1997/98. A) women, eastern Germany, B) men, eastern Germany, C) women, western Germany, D) men, western Germany.

For 1997/98, the trend segments within which the jump is tested vary considerably across the populations (Table 6; Fig 4A–4D). In models with two or three joinpoints for men and women in the west, the estimated trend segments extend well beyond Phase II (1990–2022, 1994–2020, 1993–2022). In these specifications, the estimated jumps are statistically significant and indicate a decline in suicide mortality (see Table 6).

Models with more than three joinpoints typically identify shorter segments spanning from 1993/94–2007/08. For women, these models estimate a decline (CR ≈ 0.95), but the effect is not statistically significant in eastern Germany (4–7 joinpoints) or in western Germany (5, 6 joinpoints). For women in western Germany, p-values fall between 0.05 and 0.20, indicating weaker evidence for the rejection. In the model with 7 joinpoints, a decline within a slightly different trend segment (1993–2003) is significant and even stronger in magnitude.

For men in eastern Germany, a comparable decline appears only in the model with 7 joinpoints and remains statistically insignificant. For men in western Germany, the shorter segments begin earlier (1989/90) than in the other populations (1993/94–2007/08), and the corresponding p-values indicate no significant jump is detected.

Overall, the location of the tested trend segments varied considerably with the number of joinpoints across the subpopulations. This affects the direction as well as magnitude and statistical significance of the estimated CRs. However, there are signs of a consistent methodologically induced structural break for this year pair among women in western Germany. Although similar CRs are observed for women in eastern Germany within comparable segments, they are not statistically significant. For men, either the identified segments or estimated CRs diverge from this pattern.

The existence of a general break can therefore be rejected, but a pattern of a declining jump is evident for women, particularly in western Germany.

### Hypothesis testing

Hypotheses 1a and 1b: The initial hypotheses were that in the years 1977/78 and 1997/98 there would be no methodologically induced structural break in the time series data caused by the respective ICD version change. The analyses show that the hypothesis of a methodologically induced structural break at the two year pairs could be rejected for most, but not all, populations. For women in western Germany in 1997/98, there is insufficient evidence to reject the presence of a methodologically induced structural break. Moreover, at least one substantively plausible specification identifies a statistically significant structural break for this subgroup.

Hypothesis 2: This hypothesis stated that in 1979/80 there was no methodologically induced structural break in the time series data of the GDR caused by the change in data access. The results for the most frequently occurring trend segment (mid-1970s to the second half of the 1980s) show no significant jumps for this year pair (Tables 3–6). Taking into account the threshold for beta errors (p > 0.20), it can be concluded that no methodologically induced structural break can be detected for 1979/80. Hypothesis 2 is therefore supported.

Hypothesis 3: Hypothesis 3 stated that the reorganization of official statistics in 1990/91 in eastern Germany did not lead to a methodologically induced structural break. No significant jumps were found in those trend segments that began in 1988, but they were found in those that began in 1980. However, this year pair coincides with a phase of sharp decline in suicide mortality, which is clearly linear. Furthermore, the direction of the estimated CR is inconsistent, and the fall of the Berlin Wall in 1990 was a one-off event which in some cases was associated with increased suicide mortality [24,25]. For evaluating the hypothesis, models with shorter trend segments provide a more appropriate basis for assessing potential discontinuities, and their estimated jumps are not statistically significant.

## Discussion

The first aim of the article was to merge time series from different data sources for eastern and western Germany for the period 1952–2022. These are now available.

- An initial analysis of suicide mortality trends shows the following: The development of suicide mortality can be divided into three phases for women and men in the two parts of Germany over the period analysed here. Phase I: increase in suicide mortality until the first half of the 1980s; Phase II: subsequent decline in suicide mortality until the mid-2000s; Phase III: plateau phase since the second half of the 2000s.
- Known differences in suicide mortality between eastern and western Germany (e.g., [14,17,24]) are confirmed.
- The three phases are similar in both parts of the country: although they are quantitatively different, their basic tendencies were the same.
- A downward trend has already been reported in previous studies, (e.g., [6,17,26], but only for parts of the period analysed here.
- The development of suicide mortality rates is characterized by very frequent increases and decreases and is thus highly dynamic in women and men across eastern and western Germany.
- There is no obvious evidence of methodologically induced structural breaks in the year pairs mentioned.

The second objective was to examine whether any methodologically induced structural breaks occurred in the analysed year pairs. The result of testing the three hypotheses was that there was no clear and reliable statistical evidence of a methodologically induced structural break in any of the year pairs analysed in this article. Nevertheless, there are some characteristics to be discussed relating to the respective year pairs.

### 1977/78 and 1997/98 year pairs: Change in the ICD version

There was no statistical evidence of a methodologically induced structural break consistent across all populations in either of the year pairs as postulated in hypotheses 1a and 1b. This result is supported by the fact that the Eurostat European Short List contains a category ‘Suicide and deliberate self-harm’ across ICD versions [15]. This shows that no methodologically induced break in the collection of these data is seen in the official statistics.

However, for women in Western Germany, some evidence of a jump indicating a decline is observed at both year pairs. For 1997/98, the estimated decline is statistically significant a few specifications, while others p-values range between 0.05 and 0.20, indicating weak support for a discontinuity. Comparable CRs are observed for women in Eastern Germany, although they do not reach statistical significance. A similar pattern is observed for 1977/78, where a decline is estimated across all specifications but attains statistical significance only in longer trend segments. Overall, the findings do not provide clear evidence of a methodologically induced structural break, but the recurring pattern among women warrants consideration in further research. Given a suspected general underreporting of suicide deaths [27], which begins at the time of issuing death certificates, another possible explanation is that suicide deaths among women are, at least during the transition period from the 9th to the 10th ICD-version, less often correctly recorded on death certificates. However, we are not aware of empirical study results on this matter.

There are only a few studies in the international literature that have examined the effects of ICD version changes. Using data from 71 countries for the period 1950−1999, Pearson-Nelson et al. found no overall effects on suicide mortality rates, with the exception of the switch from ICD-9 to ICD-10 [28]. For the switch from ICD-9 to ICD-10, they found an estimated decrease in the mortality rate of 0.7 per 100,000 inhabitants. Their hypothesis that improved recording and coding of causes of death with ICD-10 should lead to an increase in suicides is contrasted by the actual observed decline in suicide deaths.

Two further studies, for Italy and Norway [29] and Canada [30], concluded that ICD version changes had no effects on suicide rates. Other studies mention the topic only sporadically, if at all (e.g., [31–33]). In 2002, Bertolote & Fleischmann came to the conclusion that the category name and coding of suicide mortality are relatively stable across the different versions of the ICD [6–10,34]. There are no concrete indications of how far these results can be generalized to Germany. Our study essentially confirms the study results for Italy, Norway, and Canada. The lack of clear evidence in our findings corresponds to [28], where one may question whether a decrease in the suicide rate of 0.7 deaths per 100,000 inhabitants in a downward trend over several years can be interpreted as evidence of a methodologically induced structural break.

Overall, it is assumed that the ICD version changes did not lead to methodologically induced structural breaks in suicide mortality in either part of Germany.

A recent study concluded that the GDR suicide statistics adequately depicted the development of suicide mortality [35]. When processed by von den Driesch [18] and the Federal Statistical Office [4], all suicide mortality data that had kept secret in the GDR could apparently be incorporated without substantial gaps [6,17,18,36].

### 1979/80 year pair: Change in the data basis

Hypothesis 2 analysed the 1979/80 year pair in order to examine the possible effects of a change in the data basis. The year pair is close in time to the transition from Phase I to Phase II, during which the rise in suicide mortality ends and transitions into a decline. The application of the joinpoint-jump analysis assumes an uninterrupted general trend of the process analysed. Since this is not the case here, the results should be interpreted with caution. It should be noted that there was a profound change in the development of suicide mortality in both parts of Germany during this period, and this makes an unequivocal finding difficult. The hypothesis that the change of data source represents a methodologically induced structural break can therefore neither be confirmed nor definitively rejected.

### 1990/91 year pair: Reorganization of official statistics

For the 1990/91 year pair, the temporal variation is relatively small in all groups (which may partly reflect the larger population size in the western federal states). The overall trend can therefore be regarded as stable. The CR is not significant for women and men in the new federal states or for men in the old federal states, but it is significant for women in the old federal states (at 7 joinpoints, cf. Table 6). At the outset, it was determined that the CRs are significant for all-time series (women, men, east, west) if there is a methodologically induced structural break present, e.g., as a result of the version change. For this year pair, it can be concluded that the formulated hypothesis of a jump can be rejected. However, the beta error test shows that the associated hypotheses H1a and H1b (no methodologically induced structural break) are not fully supported by the test, because the p-values for the CRs are lower than 0.20 for women in the west. This means that the presence of a jump cannot be rejected, at least for this group. Given the strength of the decline in Phase II, on the other hand, it can be ruled out that the data contain a particularly strong methodologically induced structural break.

What is empirically striking is that the suicide mortality rates in eastern Germany were higher in 1991 than in 1990 for both women and men, which appears significant against the background of a strong overall downward trend. In the literature, this temporary increase has been linked to the social changes in the course of German reunification [6,24,25,37,38]. Wiesner and Haberland explicitly point out the impact on mental health of involuntary unemployment, which occurred on a large scale after 1990 [24].

The interruption of the trend is therefore more likely to be due to the consequences of social change and the transformation process in eastern Germany.

### Summarizing assessments

Table 7 summarizes the criteria for identifying a methodologically induced time-series break, as presented in the methods section, and the respective results. Column 1 lists the year pairs, and column 2 the number of subpopulations (east/west, female/male). This forms the basis for further tests of the respective trend segments (criterion 1). This is used to carry out further tests for the respective trend segment (criterion 1). Column 3 shows the two most frequently identified trend segments from Tables 3–6. Column 4 shows how often each trend segment occurred in the respective year pair for all subpopulations. For example, for the 1977/78 year pair, the trend segment 1952–1981–85 occurs 15 times in all models (with 2–7 joinpoints each) and the trend segment 1975–77–1981–87 occurred 8 times. Thus, in 23 out of 24 models (6 models with different joinpoints x sex x east/west) two trend segments were identified. One model (model with 7 joinpoints, men, west: 1967–1985) could not be assigned to the two most frequently found trend segments. In total 11 models could be assigned to the two most common trend segments. Columns 5 and 6 show the direction of the jumps and the CRs of the trend segments (<1.0 or >1.0). Columns 7 and 8 characterize the number of joinpoints and the approximate duration for the respective trend segments. Columns 9 and 10 contain the results of the test for significance of the CRs (criterion 3) and for beta errors (criterion 4). Column 11 provides the overall assessment.

**Table 7:** Examination of the criteria based on the results of the models with 7 joinpoints.

Year pair	Number of groups	Trend segments: period	Trend segments: number per period	Jump	CRs of the trend segments	Models with number JP	Average length of trend segments (in years)	Significance alpha	Significance beta	Overall assessment
1	2	3	4	5	6	7	8	9	10	11
1977/78	4	1952 to 1981−85	15	↘	15 out of 15: < 1.0	2-5	30	n.s.: 12 out of 15; p < 0.05: 3 out of 15	.05 < p < .20: 6 out of 15	No consistent break
		1975−77–1981−87	8	↗	6 out of 8: > 1.0	4-7	10	n.s.: 8 out of 8;	.05 < p < 0.20: 0 out of 8	
			1^a)^	.	.	.	.	.	.	
1979/80	2	1952 to 1982−85	5	↘	5 out of 5: < 1.0	2-5	30	n.s.: 3 out of 5; p < 0.05: 2 out of 5	.05 < p < 0.20: 1 out of 5	No consistent break
		1975/76–1987/88	6	↗	6 out of 6: >1.0	4-7	10	n.s.: 6 out of 6	.05 < p < 0.20: 0 out of 6	
			1^a)^	.	.	.	.	.	.	
1990/91	2	1980−83 until 2006/07	6	↘	6 out of 6: <1.0	2-5	25	n.s.: 1 out of 6; p < 0.05: 5 out of 6	.05 < p < 0.20: 1 out of 6	No consistent break
		1988 to 1993/94	6	↗	4 out of 6: >1.0	4-7	6	n.s.: 6 out of 6	.05 < p < 0.20: 0 out of 6	
1997/98	4	1980−85–2005−07	6	↗	6 out of 6: >1.0	2-5	25	n.s.: 6 out of 6	.05 < p < 0.20: 4 out of 6	No consistent break
		1993/94–2003−08	9	↘	8 out of 9: <1.0	4-7	15	n.s.: 8 out of 9 p < 0.05: 1 out of 9	.05 < p < 0.20: 2 out of 9	
			9 ^b)^	.	.	.	.	.	.	

Source: own compilation.

a)another trend segment that does not fit into most frequent periods.

b)further trend segments that neither conform to the most frequent periods nor form a coherent period group of their own (see Table 6).

For all year pairs, it can be observed that the jumps for long time periods often point in the opposite direction compared to the jumps for short periods. With few exceptions, the CRs are generally not significant. In none of the year pairs are the CRs significant for all populations.

The results for men in eastern Germany in 1990/91 deserve a special consideration. In models with 2–5 joinpoints, the CR is less than 1, although the empirical values show an increase from 1990 to 1991. This increase temporarily interrupted the general trend of decline during Phase II. This phenomenon, which also occurred among women in eastern Germany, but to a lesser extent, can be attributed to the major social changes in eastern Germany in 1989/90/91. In the models with the higher number of joinpoints (women 4–7 joinpoints; men 5–7 joinpoints), the CRs turn into value ranges greater than 1, with p-values outside the interval of 0.05 to.20. The hypothesis of no significant jump can therefore not be rejected.

In summary, based on the results of the step-by-step procedure, there is no evidence of a methodologically induced structural break consistent for all populations at any of the analysed year pairs. However, for the 1977/78 year pair, a statistically significant decline is observed in three out of 15 models and in further six models, p-values of between 0.05 and 0.20 are observed. For the 1997/98 year pair, one model shows significance and two additional models out of nine yield p-values between 0.05 and 0.20. Detailed inspection of the results section (see above, Table 3 and Table 6) indicates that these patterns of a jump are primarily observed for women. This finding deserves further investigation in future research.

The quality of suicide-mortality data is often discussed. In general, official statistics are assumed to underreport cases (see, for example, the systematic review [27]). This underreporting is unlikely to systematically influence the trends in suicide mortality.

The reliability of the data from the official statistics of the GDR is also subject to debate. The mandated secrecy of the official suicide statistics suggests that the GDR leadership regarded this data as very problematic and sensitive [18,35]. However, there is no evidence of any deliberate falsification of the data [18]. According to Grashoff, the continued secrecy surrounding suicide statistics indicates that the data were not falsified [35]: falsified figures would not have required secrecy.

## Summary

The empirical results show that suicide mortality was highly dynamic in both parts of Germany, with frequent fluctuations in rates of increase and decrease. Despite elaborate and complex analyses, no indications of methodologically induced structural breaks were found for men. For women, the findings are inconclusive regarding ICD version changes, where weak evidence of a break was observed. For both sexes, no breaks were detected for the transition of the GDR statistical system to that of the FRG.

Overall, the compiled long time series for eastern and western German women and men are largely consistent, but caution is warranted when interpreting trends for women encompassing periods of ICD-code changes, although any methodological effects are likely within the range of typical annual fluctuations (±5%).
